# Unveiling rare and severe complications of respiratory viruses: A diverse case series of influenza A, influenza B, and Covid-19

**DOI:** 10.5339/qmj.2024.qitc.16

**Published:** 2024-03-25

**Authors:** Sreethish Sasi, Jouhar Kolleri, Fatma Ben Abid, Maliha Thapur, Arun P Nair, Muna Al-Maslamani

**Affiliations:** Infectious Diseases Division, Department of Internal Medicine, Communicable Diseases Center, Hamad Medical Corporation, Doha, Qatar Email: ssasi7@hamad.qa; Department of Clinical Imaging, Hamad Medical Corporation, Doha, Qatar

**Keywords:** AHEM, Influenza-A, Influenza-B, Covid-19, Tamponade

## Background

A majority of cardiac complications of influenza are related to influenza A.^1^ Coxsackie B, parvovirus B-19, HHV-6, and cytomegalovirus are the most frequently implicated causes of viral myocarditis-related cardiac tamponade.^2^ The viral etiology of some neuro-infections is well described in the literature, especially those related to neurotropic viruses such as poliovirus, coxsackievirus, and enterovirus-71.^3^ This case series explores rare and severe complications associated with respiratory viruses, influenza A, influenza B, and Covid-19.

## Case Summaries

**Figure 1.**
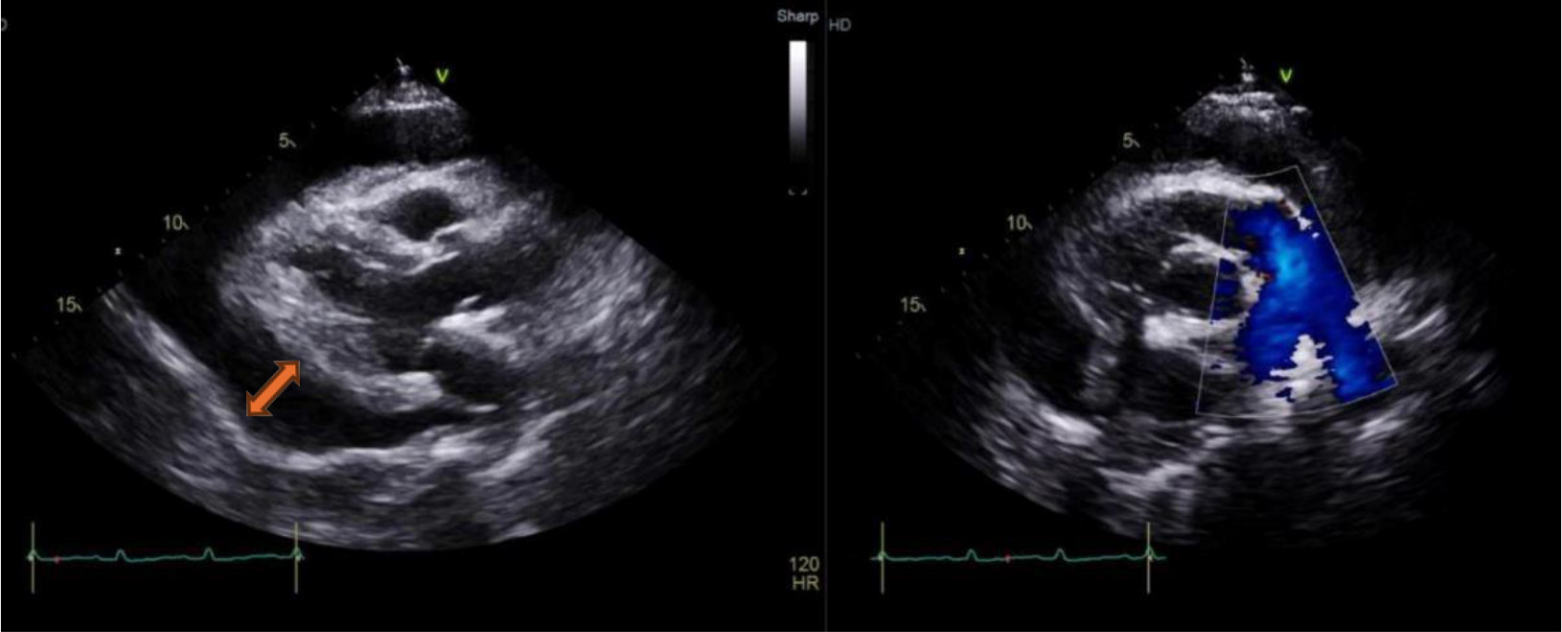
Transthoracic echocardiogram showing a large pericardial effusion (orange arrow) with impending cardiac tamponade.

**Case 1:** A 22-year-old woman with influenza B presented with acute dyspnea and was found to have cardiac tamponade (Figure 1). The patient responded well to treatment and was discharged after one week.

**Figure 2.**
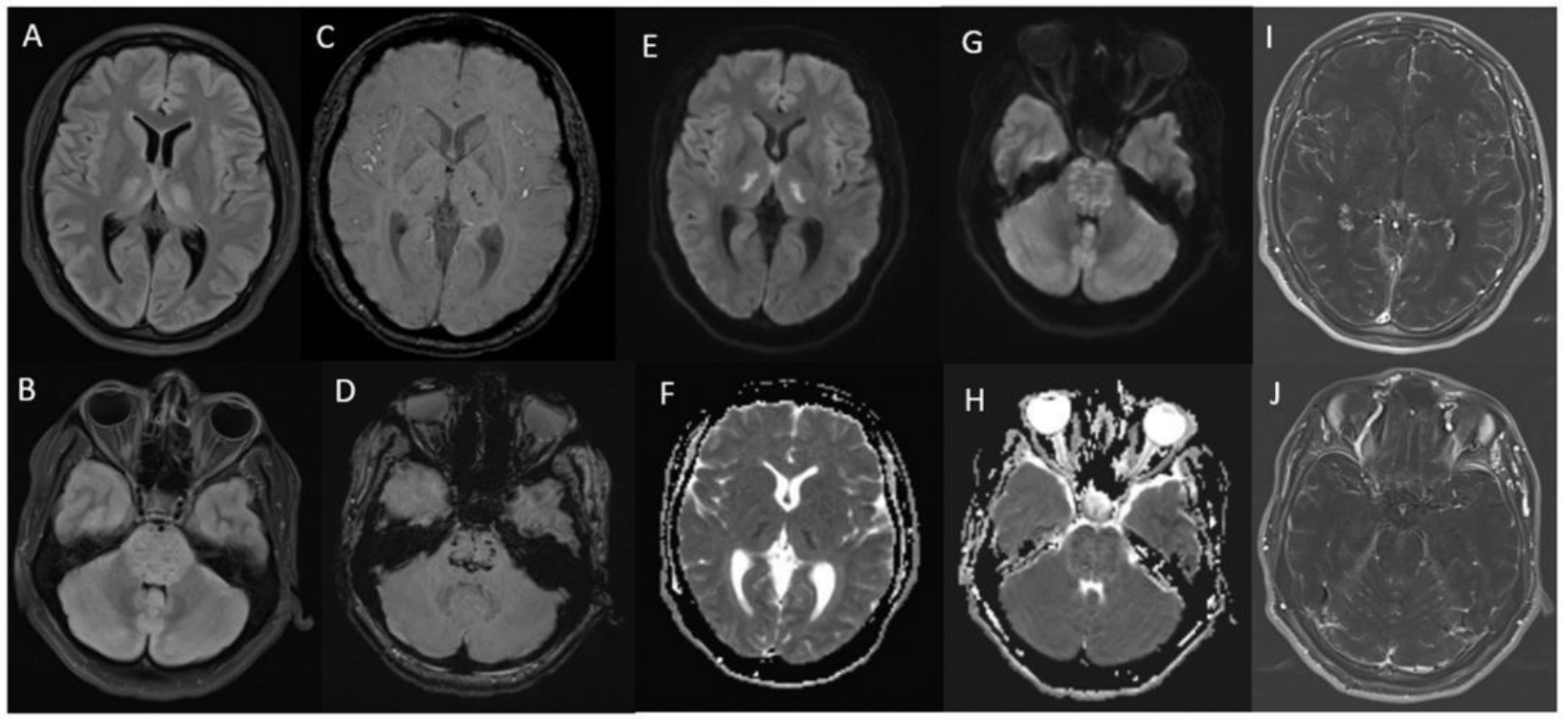
MRI brain with contrast showing acute hemorrhagic encephalomyelitis (case 2). (A,B) Axial fluid attenuated inversion recovery (FLAIR), (C,D) Susceptibility weighted imaging (SWI), (E,G) Diffusion weighted imaging (DWI), (F,H) Apparent Diffusion coefficient (ADC) sequences, (I,J) T1 post-contrast subtraction images showing bilateral almost symmetrical abnormal diffusion restriction in the thalami and swollen pons, corresponding high FLAIR signal intensity with extensive micro hemorrhages on SWI and diffuse extensive bilateral symmetrical leptomeningeal enhancement involving both cerebellar and cerebral hemispheres.

**Case 2:** A 27-year-old woman with influenza A presented with flu-like symptoms, headache, and seizures. Further investigation revealed acute hemorrhagic encephalomyelitis (AHEM) (Figure 2), a rare neurological complication associated with influenza A. Despite initial stabilization, the patient’s condition deteriorated, and she passed away.

**Figure 3.**
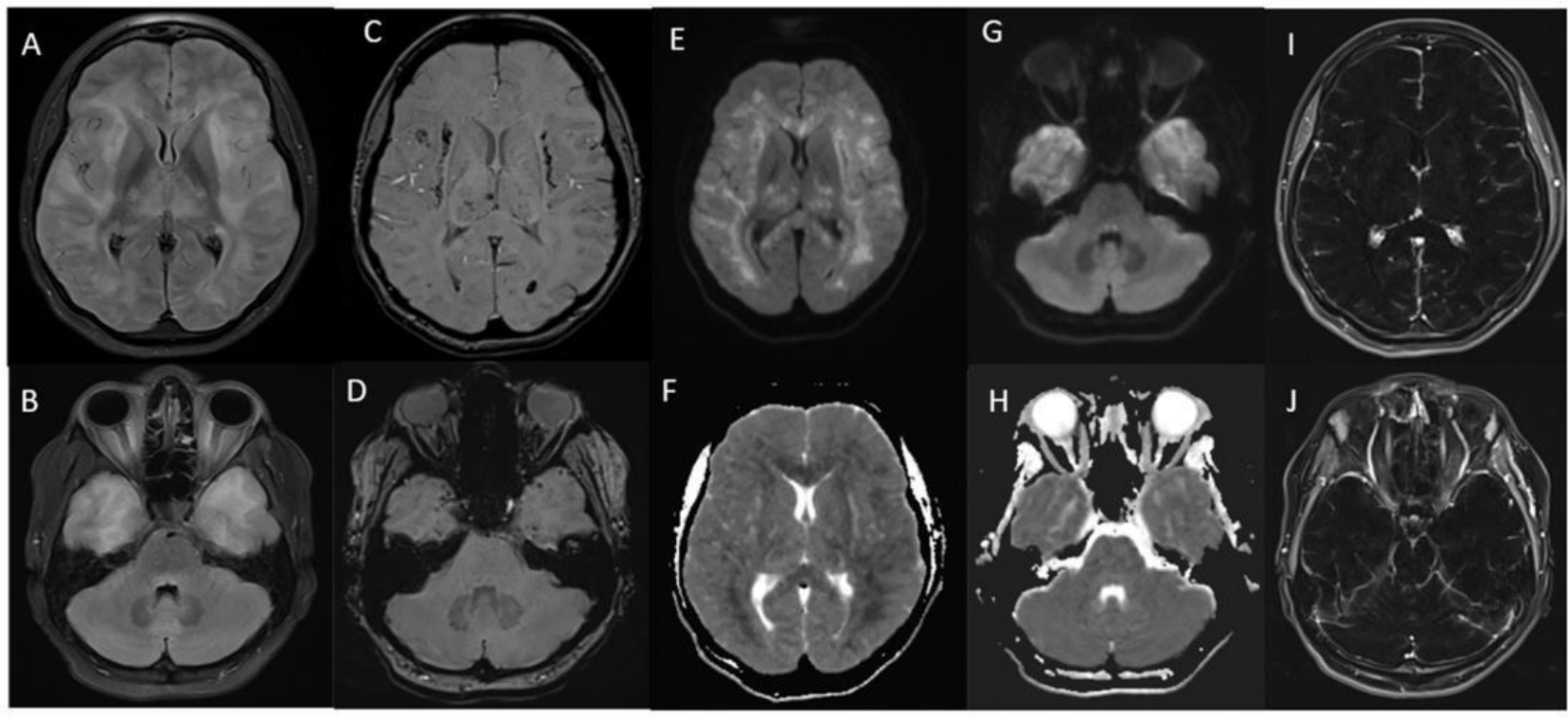
MRI brain with contrast showing acute hemorrhagic encephalomyelitis (case 3). (A,B) Axial fluid attenuated inversion recovery (FLAIR), (C,D) Susceptibility weighted imaging (SWI), (E,G) Diffusion weighted imaging (DWI), (F,H) Apparent Diffusion coefficient (ADC) sequences, (I,J) T1 post contrast subtraction images showing diffuse brain swelling, increased intra-cranial pressure, scattered white matter, thalamus and brain-stem diffusion restriction, increase FLAIR signals and micro hemorrhages on SWI image along with bilateral leptomeningeal enhancement.

**Case 3:** A 30-year-old woman with Covid-19 presented with fever, headache, and seizures. Imaging showed signs of AHEM (Figure 3), but unlike other similar cases, the patient had only mild respiratory symptoms and no evidence of infection in the cerebrospinal fluid. The patient passed away after a week of hospitalization.

## Conclusion

This case series highlights the importance of recognizing and understanding the diverse complications that can arise from respiratory viruses. It emphasizes the need for clinicians to be aware of atypical presentations, including both cardiac and neurological sequelae. By expanding our knowledge of these complications, clinicians can improve early recognition and tailored management, leading to better patient outcomes.

## Conflict of Interest

The authors have no conflicts of interest to declare.
